# 2662. Efficacy of Sitafloxacin and Doxycycline Combination Therapy as a Salvage Treatment for Refractory *Mycoplasma genitalium* Infection

**DOI:** 10.1093/ofid/ofad500.2273

**Published:** 2023-11-27

**Authors:** Naokatsu Ando, Daisuke Mizushima, Misao Takano, Morika Mitobe, Kai Kobayashi, Hirofumi Miyake, Hiroaki Kubota, Jun Suzuki, Shinichi Oka, Hiroyuki Gatanaga

**Affiliations:** National Center for Global Health and Medicine, Shinjuku-ku, Tokyo, Japan; National Center for Global Health and Medicine, Shinjuku-ku, Tokyo, Japan; National Center for Global Health and Medicine, Shinjuku-ku, Tokyo, Japan; Tokyo Metropolitan Institute of Public Health, Shinjuku-ku, Tokyo, Japan; Tokyo Metropolitan Institute of Public Health, Shinjuku-ku, Tokyo, Japan; Tokyo Metropolitan Institute of Public Health, Shinjuku-ku, Tokyo, Japan; Tokyo Metropolitan Institute of Public Health, Shinjuku-ku, Tokyo, Japan; Tokyo Metropolitan Institute of Public Health, Shinjuku-ku, Tokyo, Japan; National Center for Global Health and Medicine, Shinjuku-ku, Tokyo, Japan; National Center for Global Health and Medicine, Shinjuku-ku, Tokyo, Japan

## Abstract

**Background:**

The prevalence of resistant strains of *Mycoplasma genitalium* has increased over the past few decades, leading to limited treatment options. This study aimed to evaluate the efficacy of sitafloxacin and doxycycline combination therapy as a salvage treatment for refractory rectal and urogenital *M. genitalium* infections.

**Methods:**

Men who have sex with men (MSM) diagnosed to have *M. genitalium* infection from urine samples or rectal swabs using a PCR assay were evaluated and administered sitafloxacin 200 mg and doxycycline 200 mg daily for 21 days. Each sample was tested for *23rRNA*, *parC*, and *gyrA* mutations before and if a failure after the treatment.

**Results:**

A total of 27 MSMs were included. The median age was 33 years (range 21-47), and 33.3% (9/27) were people with HIV(PWH) (Table). Among PWH, the median CD4 count was 642, and all were virally suppressed with antiretroviral therapy. Positive samples included 22 rectal and 5 urine specimens. The time to test of cure(TOC) was 14 days (range 0-227). In the two failure cases 9 and 20, the TOC was conducted too early, and it was rechecked after 21 days following treatment completion; both rechecked TOCs were positive. *ParC* mutations were detected in 100%(23/23) of the samples, *gyrA* mutations in 66.7%(16/24) and ,macrolide-resistance associated mutations in 100%(22/22). Among the *parC* mutations, 95.7%(22/23) was G248T(S83I), while 4.3%(1/23) was G259T(D87Y). The overall cure rate was 81.5%. The cure for rectal infection was 81.8%(18/22), while the cure rate for urogenital infection was 80%(4/5). No significant difference was observed between the anatomical sites (p=0.924). The cure rate for strains harboring *parC* mutation and wild-type in *gyrA* was 85.7% (6/7), whereas the cure rate for the strain harboring *parC* mutation and *gyrA* mutation was 73.3%(11/15). No significant difference was found regardless of *gyrA* mutations (p=0.519). Additionally, no significant difference was observed in the cure rate between TOC < 21 days and >= 21 days (p=0.964).

Cases of Mycoplasma genitalium treatment and antimicrobial-resistance profiles in participants before and after treatment with the combination therapy

**Figure:**
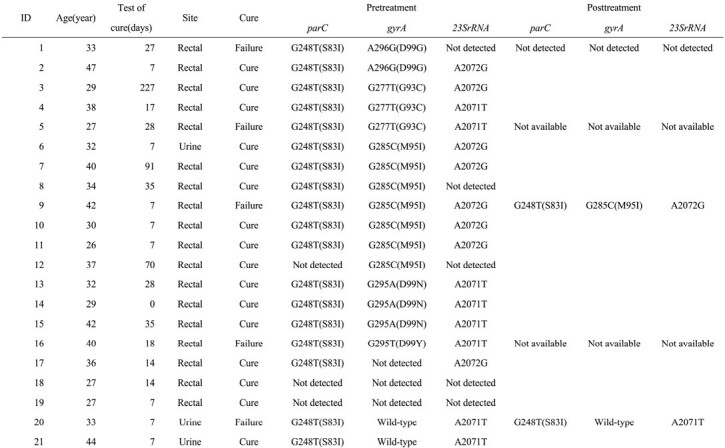

**Conclusion:**

The combination therapy showed high efficacy of 81.5% for highly resistant *M. genitalium* infection and can be considered as a salvage treatment option for refractory cases.

**Disclosures:**

**All Authors**: No reported disclosures

